# A comparison between the effects of drug costs and share of family income on drug costs in determining drug price

**DOI:** 10.1097/MD.0000000000026877

**Published:** 2021-08-06

**Authors:** Xiaobei Dong, Chi Chun Steve Tsang, Anoop Kotian, Jason Zeng, Michael Tran, Junling Wang

**Affiliations:** a Department of Clinical Pharmacy and Translational Science, College of Pharmacy, University of Tennessee Health Science Center, 881 Madison Avenue, Room 214, Memphis, TN; b Department of Clinical Pharmacy and Translational Science, College of Pharmacy, University of Tennessee Health Science Center, 881 Madison Avenue, Room 212, Memphis, TN; c College of Pharmacy, University of Tennessee Health Science Center, 881 Madison Avenue, Memphis, TN; d Department of Clinical Pharmacy and Translational Science, College of Pharmacy, University of Tennessee Health Science Center, 881 Madison Avenue, Room 221, Memphis, TN.

**Keywords:** affordability, drug costs, drug price, health care access, income

## Abstract

High health care and medication expenditures pose a financial burden on Americans seeking care. It is imperative to determine the role of affordability in influencing access to health care and medications.

To investigate the association between financial burden and health care access by comparing the effects of absolute and relative financial burdens, measured by total health care/medication expenditure (Expenditure) and health care/medication expenditure as a share of annual family income (Expenditure Share), respectively.

Delay in receiving health care services and delay in obtaining prescription medications.

A cross-sectional analysis of the 2017 Medical Expenditure Panel Survey using multivariate logistic regressions with Expenditure and Expenditure Share variables standardized to facilitate comparison.

While both absolute and relative financial burdens were found to be positively associated with the outcomes, the relative measure had a significantly higher association that was about twice as much as the absolute one. For the outcome of delay in getting health care, the standardized odds ratios (OR) for health care expenditure and health care expenditure as a share of family income were 1.13 (95% confidence interval [CI] = 1.09–1.18) and 1.25 (95% CI = 1.20–1.32), respectively. For the outcome of delay in getting medications, the standardized OR for medication expenditure and medication expenditure as a share of family income were 1.11 (95% CI = 1.08–1.15) and 1.23 (95% CI = 1.18–1.29), respectively.

The study illustrated the importance of including income in policy considerations intended to balance value, access, and affordability. Specifically, income should be included in measures assessing the value of medications.

## 1 Introduction

Rising health care costs has been an issue entrenched in the U. S. for over a decade,^[1]^ garnering widespread public attention in 2010 with the enactment of the Patient Protection and Affordable Care Act. Despite government interventions, health care spending remained unchecked. When compared with 10 other high-income countries, the U. S. topped not only the percentage of gross domestic product spent on health care, but also health expenditure per capita. The latter was nearly twice as much as the mean of all 11 countries.^[2]^ While all types of health care incur cost, medications were found to be the largest driver.^[3]^ The U. S. pharmaceutical spending per capita was a staggering $1,443, compared to a range of $466 to $939 in other high-income countries.^[2]^ Drug expenditure was not only high but also increasing at an accelerated speed in recent years. The prescription medication spending grew by 20% from 2013 to 2015, almost doubling the increase rate of 11% in overall health care spending.^[4]^ Moreover, high medication expenditure did not come with high health service utilization. While Americans on average spent over 200% more on primary care prescription drugs than their counterparts in comparable countries, they received 12% less of therapeutic treatment.^[5]^

Such high health care and medication expenditures pose a financial burden on Americans seeking needed care, and the burden has been on the rise since the turn of this century.^[6]^ Compared with its high-income peers, the U. S. had the highest percentage of adults with cost-related access barriers, examples of which included forgone doctor's visits, medical treatment, or prescription medications due to cost.^[7]^ Additionally, the barriers have been compounded in recent years by the movement in private insurance towards so-called “consumer-directed”health plans featuring high deductibles.^[8]^ Increased out-of-pocket (OOP) expenditure not only had an immediate impact on health care access but could also affect outcomes further down the stream such as modified health service utilization patterns, reduced medication adherence and, ultimately, adverse health outcomes.^[4,9]^ Healthy People 2030 has recognized health care access as one of the national priorities over the next decade, with addressing cost-related barriers identified as one of the most effective approaches to ensure access.^[10]^

However, such barriers were higher for some than others. Not all financial burdens were incurred equal. Previous studies indicated that the effect of expenditure on health care differed by income level. For low-income families, even seemingly small OOP expenditure could become a heavy financial burden.^[11]^ Compared to higher-income Medicare families, those with an income up to 250% of the Federal Poverty Level were found more likely to have cost-related burden.^[3]^ Likewise, income-related disparities were found in delayed or forgone care for families with children. Those reported having unmet health care needs were more likely to have lower income.^[12]^

As illustrated earlier, expenditure on drugs was found to be the largest driver of financial burden. The challenge of ensuring health care access while keeping it affordable, therefore, cannot be overcome without tackling the ever-increasing medication expenditure. The price of drugs is based on their assessed value.^[13]^ Thus, it is imperative to have a valuing measure that takes into account the need of accessibility and affordability. The purpose of this study is to provide empirical evidence that can be used to inform the development of such a measure. In particular, this study sought to further investigate the association between financial burden and health care access by comparing the effects of absolute and relative financial burdens, measured by total health care/medication expenditure and health care/medication expenditure as a share of annual family income, respectively.

## 2 Methods

### 2.1 Data source and study sample

This study was a retrospective cross-sectional analysis of the 2017 Medical Expenditure Panel Survey – Household Component (MEPS-HC). MEPS-HC is a nationally representative survey of the U. S. civilian noninstitutionalized population. It collects data from households and their members on a wide range of subjects including health expenditure, access to care, health service utilization, health insurance coverage, income, health status, and demographic characteristics.^[14]^

The study sample included respondents with a family income larger than zero and an annual health care expenditure no more than 100% of the family income. Individuals with negative or zero family income as well as those who spent more than 100% of their family income on health care were excluded, because one might expect that the health care consumption behaviors of such individuals differed from those who utilized health care within their budget constraints. The inclusion criteria were applied to reduce the heterogeneity of the study sample, thereby allowing for a more precise measure of the affordability of health care/medications.

### 2.2 Outcome variables

Two dummy outcome variables measuring access to care were constructed from MEPS-HC variables that contained responses to questions asking whether respondents experienced delay in getting medical care, dental care, or prescription medications in the past 12 months.^[15]^ One outcome variable captured delay in receiving health care services in general, with the value of one indicating a respondent experiencing delay in at least one of the 3 types of aforementioned health care services and the value of zero indicating no delay. A separate outcome variable was constructed for a distinct analysis of factors associated with medication access, with the value of one indicating delay in obtaining prescription medications and the value of zero indicating no delay.

### 2.3 Theoretical framework

This study used the Gelberg-Andersen Behavioral Model for Vulnerable Populations as its theoretical framework.^[16]^ Factors potentially associated with the outcome variables were selected as covariates based on the predisposing, enabling, and need components of the model. The predisposing domain, which includes demographic and social structure characteristics, was measured by age, gender (male and female), race/ethnicity (non-Hispanic Whites, non-Hispanic Blacks, Hispanics, non-Hispanic Asians, and non-Hispanic other/multiple race), marital status (married and unmarried), and education level (less than or equal to high school and greater than high school). The enabling domain, which is comprised of personal and community resources, was measured by health care/medication total expenditure, health care/medication expenditure as a share of family income, insurance type (private, public, and none), poverty category (poor, low, middle, and high income), and census region (Northeast, Midwest, South, and West). The need domain, which encompasses perceived and evaluated risk in health, was measured by respondents’ self-perceived health status (excellent, very good, good, fair, and poor).

### 2.4 Statistical analyses

To assess the effects of total health care/medication expenditure (Expenditure) versus health care/medication expenditure as a share of annual family income (Expenditure Share) on delay in obtaining health care/medications, ceteris paribus, multivariate logistic regression analyses were conducted. To facilitate the comparison of the effects, the Expenditure and Expenditure Share variables were standardized prior to regressing the corresponding outcome on them. The standardization was done by first calculating the deviation of the variable from its mean and then dividing the difference by its standard deviation. This process transformed both variables to standard scores with a mean of zero and a standard deviation of one. By setting both variables on the same scale, the standardization allowed for a direct comparison of effects between the 2. Two otherwise identical logistic regression models were then fit for each outcome, with 1 model using standardized Expenditure and the other using standardized Expenditure Share as the independent variable of interest. Non-overlapping confidence intervals (CI) of the 2 standardized variables would indicate a statistically significant difference between the Expenditure and Expenditure Share effects. All analyses were conducted using SAS 9.4,^[17]^ with statistical significance level set *a priori* at 0.05. The Institutional Review Board at the corresponding author's institution approved the study (approval number #20–07753-NHSR).

## 3 Results

The study sample included 21,508 individuals needing health care services and 21,469 individuals needing prescription medications. Their demographic and socio-economic characteristics are presented in Table 1. The distributions of characteristics were similar across both study cohorts. The individuals were more likely to be female and non-Hispanic Whites, and less likely to have education level greater than high school. They were also more likely to have private insurance and high income, reside in the South census region, and perceive their health status as very good. Among the health care services cohort, the average annual health care expenditure and health care expenditure as a share of family income were $3,833.68 and 8%, respectively. By comparison, the prescription medication cohort on average had an annual medication expenditure of $992.49 and such expenditure accounted for 2% of family income.

**Table 1:** Characteristics of the study sample by cohort in need of health care services and prescription medications (number and % unless otherwise specified).

	HC (N = 21,508)		MED (N = 21,469)	
Characteristics	Number	%	Number	%
Predisposing factors				
Age, mean (SD)	46.56 (18.12)		46.57 (18.13)	
Male	10,154	47.21	10,133	47.20
Race/Ethnicity				
Non-Hispanic Whites	10,489	48.77	10,470	48.77
Non-Hispanic Blacks	3463	16.10	3449	16.07
Hispanics	5493	25.54	5489	25.57
Non-Hispanic Asians	1411	6.56	1409	6.56
Non-Hispanic Other/Multiple Race	652	3.03	652	3.04
Married	10,785	50.14	10,768	50.16
Education > High School	7720	35.89	7707	35.90
Enabling Factors				
Expenditure, mean (SD)	3833.68 (7902.81)		992.49 (3727.74)	
Expenditure share, mean (SD)	0.08 (0.16)		0.02 (0.07)	
Insurance type				
Private	13,845	64.37	13,816	64.35
Public	5359	24.92	5350	24.92
No insurance	2304	10.71	2303	10.73
Poverty Category				
Poor	3594	16.71	3593	16.74
Low income	3259	15.15	3259	15.18
Middle income	6608	30.72	6582	30.66
High income	8047	37.41	8035	37.43
Census regions				
Northeast	3402	15.82	3397	15.82
Midwest	4415	20.53	4401	20.50
South	8152	37.90	8134	37.89
West	5539	25.75	5537	25.79
Need factor				
Self-perceived health status				
Excellent	5324	24.75	5311	24.74
Very good	7194	33.45	7186	33.47
Good	6418	29.84	6406	29.84
Fair	2127	9.89	2122	9.88
Poor	445	2.07	444	2.07

Table 2 presents the results of multivariate logistic regression analysis by outcome. For each outcome, the left column reports estimates from the model using Expenditure as the independent variable of interest and the right column presents estimates from the model using Expenditure Share. While both Expenditure and Expenditure Share were found to be positively associated with the corresponding outcome, the latter's association was approximately twice as much as the former's and the difference was statistically significant. Specifically, for the outcome of delay in getting health care, the standardized odds ratios (OR) for health care expenditure and health care expenditure as a share of family income were 1.13 (95% CI = 1.09–1.18) and 1.25 (95% CI = 1.20–1.32), respectively. For the outcome of delay in getting medication, the standardized OR for medication expenditure and medication expenditure as a share of family income was 1.11 (95% CI = 1.08–1.15) and 1.23 (95% CI = 1.18–1.29), respectively.

**Table 2:** Multivariate logistic regression analysis on factors associated with delay in getting HC and MED.

	Delay in HC		Delay in MED	
Characteristics	OR (95% CI)	OR (95% CI)	OR (95% CI)	OR (95% CI)
Predisposing factors				
Age	1.00 (1.00–1.01)	1.00 (1.00–1.01)	1.00 (1.00–1.01)	1.00 (1.00–1.01)
Male	0.79 (0.69–0.91)	0.80 (0.70–0.92)	0.82 (0.68–1.00)	0.84 (0.69–1.02)
Race/Ethnicity				
Non-Hispanic Whites	Reference	Reference	Reference	Reference
Non-Hispanic Blacks	0.78 (0.64–0.94)	0.80 (0.66–0.97)	0.81 (0.60–1.11)	0.83 (0.60–1.13)
Hispanics	0.49 (0.39–0.63)	0.52 (0.41–0.66)	0.36 (0.25–0.51)	0.37 (0.26–0.53)
Non-Hispanic Asians	0.40 (0.28–0.56)	0.40 (0.28–0.58)	0.21 (0.10–0.42)	0.20 (0.10–0.42)
Non-Hispanic Other/Multiple Race	0.88 (0.64–1.22)	0.88 (0.64–1.21)	0.88 (0.51–1.50)	0.87 (0.49–1.52)
Married	0.62 (0.54–0.72)	0.66 (0.57–0.76)	0.60 (0.48–0.75)	0.63 (0.51–0.79)
Education > High School	1.32 (1.15–1.52)	1.32 (1.15–1.51)	1.45 (1.18–1.79)	1.45 (1.18–1.79)
Enabling Factors				
Expenditure	1.13 (1.09–1.18)		1.11 (1.08–1.15)	
Expenditure share		1.25 (1.20–1.32)		1.23 (1.18–1.29)
Insurance type				
Private	Reference	Reference	Reference	Reference
Public	1.10 (0.93–1.30)	1.08 (0.91–1.28)	1.22 (0.93–1.61)	1.17 (0.88–1.56)
No insurance	1.30 (1.00–1.68)	1.40 (1.09–1.81)	1.67 (1.20–2.31)	1.80 (1.30–2.51)
Poverty category				
Poor	Reference	Reference	Reference	Reference
Low income	0.92 (0.72–1.18)	1.02 (0.80–1.32)	0.68 (0.49–0.95)	0.77 (0.55–1.07)
Middle income	0.93 (0.75–1.14)	1.15 (0.93–1.42)	0.89 (0.64–1.25)	1.08 (0.76–1.54)
High income	0.75 (0.58–0.98)	1.04 (0.81–1.36)	0.65 (0.45–0.94)	0.86 (0.59–1.25)
Census regions				
Northeast	Reference	Reference	Reference	Reference
Midwest	1.16 (0.93–1.43)	1.16 (0.94–1.44)	1.24 (0.91–1.69)	1.21 (0.89–1.66)
South	0.97 (0.78–1.20)	0.97 (0.78–1.21)	1.03 (0.77–1.37)	1.01 (0.75–1.35)
West	1.38 (1.11–1.72)	1.40 (1.12–1.74)	1.27 (0.89–1.82)	1.29 (0.90–1.85)
Need factor				
Self-perceived health status				
Excellent	Reference	Reference	Reference	Reference
Very good	1.75 (1.42–2.16)	1.74 (1.41–2.15)	1.89 (1.29–2.77)	1.86 (1.27–2.73)
Good	2.09 (1.73–2.52)	2.05 (1.69–2.47)	2.44 (1.69–3.51)	2.35 (1.62–3.40)
Fair	3.94 (3.07–5.04)	3.68 (2.86–4.74)	5.91 (3.98–8.78)	5.36 (3.58–8.04)
Poor	5.58 (4.01–7.77)	4.93 (3.48–6.97)	8.93 (5.38–14.84)	7.88 (4.66–13.35)

Other individual characteristics were also found to be significantly associated with the outcomes. The associations were generally similar across both outcomes. For example, estimates from the right column for the outcome of delay in getting health care indicated the following: Such delay was negatively associated with male gender (OR = 0.80; 95% CI = 0.70–0.92). Non-Hispanic Blacks (OR = 0.80; 95% CI = 0.66–0.97), Hispanics (OR = 0.52; 95% CI = 0.41–0.66), and non-Hispanic Asians (OR = 0.40; 95% CI = 0.28–0.58) were less likely to have delay in receiving health care compared to non-Hispanic Whites. The outcome was also negatively associated with married status (OR = 0.66; 95% CI = 0.57–0.76) and positively associated with education level greater than high school (OR = 1.32; 95% CI = 1.15–1.51). Uninsured patients were more likely to have delay than those having private insurance (OR = 1.40, 95% CI = 1.09–1.81). Compared to patients with excellent self-perceived health, patients with a worse self-perceived health were more likely to experience delay. The ORs ranged from 1.74 (95% CI = 1.41–2.15) to 4.93 (95% CI = 3.48–6.97) and increased as the level of self-perceived health decreased.

## 4 Discussion

While both absolute and relative financial burdens were found to be positively associated with a delay in receiving health care/medications, the analysis revealed that the relative measure had a significantly higher association that was about twice as much as the absolute one. While an OR estimate less than 1.5 is classified as a “weak” association, the results are still statistically significant.^[18]^ The immediate implication is that efforts to evaluate access barriers should focus on how much consumers spent on health care/medication in relation to their income, not merely how much was spent, which may be misleading. Given that drug expenditure accounts for a substantial portion of health care cost, a broader implication is that income should be included in determining the value of drugs, which is used as the basis for calculating drug prices. When value-based pricing is disconnected from income, drugs may cost beyond the budget limit of lower-income population, who may subsequently be faced with increased unmet health care needs due to unaffordable medications.

In fact, concerns have been raised around the efficacy of cost-effectiveness analysis (CEA), the current value assessment methodology intended to provide scientific evidence to ensure medical treatments, including drugs, are valued in a way that is fair for both manufacturers and consumers.^[19]^ One metric in the CEA model that has been scrutinized is quality-adjusted life year (QALY).^[20]^ Touted by the Institute for Clinical and Economic Review as the “gold standard” for cost-effectiveness measurement, QALY evaluates the degree to which a medical treatment lengthens or improves the lives of patients.^[19]^ However, the current QALY threshold amounts to $150,000,^[19]^ which is more than double of the U. S. median household income. Medications valued by QALY and priced accordingly would be out of reach for lower-income families. When the entire nation continued to spend more than it could afford on health care, its economy would suffer from a structural deficit with an oversized health care sector stifling everything else. Concerns have also been voiced about related measurement issues such as the way willingness to pay was gauged in survey research. Typically, the sampling frame was comprised of individuals with higher income levels, whose endorsement of QALY may not be representative of the entire population.^[20]^

Without accounting for income, the current valuing method leaves out a significant proportion of the population who have lower socioeconomic status and therefore are more likely to have financial burdens and unmet health care needs. If access and affordability hinge upon the fair pricing of medications, the current method may fall short of improving either and likely perpetuate health disparity. High medication and health care costs constitute financial burdens on not just consumers but also governments because more resources would be needed to care for a population with suboptimal health outcomes induced by unmet health care needs. Having to grapple with rising health care costs and budget constraint is not a challenge unique to the U. S. countries in Europe and Australia explored alternatives to CEA and saw an increased adoption of multiple criteria decision analysis, which includes additional elements of value such as budget impact.^[21]^ The U. S. government's response to the same challenge over the past decade has been a shift from volume-based to value-based care.^[22]^ Major legislations passed in this regard included the Affordable Care Act in 2010 and the Medicare Access and CHIP Reauthorization Act in 2015, changing the reimbursement methods to value-based for hospitals and Medicare physicians, respectively.^[7]^ As value continues to be the focal point of national health care policies targeting access and affordability, it is crucial to incorporate income in the discourse so that the policies do not leave behind the lower-income population, those most vulnerable to high health care cost.

This study has several limitations. First, MEPS-HC is self-reported data. There might be potential bias built in from errors in recollection. For instance, respondents might have overestimated or underestimated their income or health care need. Second, the income variable from MEPS represents total family income. A more accurate measure would be disposable family income. Third, the MEPS expenditure variable collapses payments from different sources including out of pocket and insurers. An alternative expenditure measure with a breakdown that separates OOP payment from other sources of payment would be helpful in further exploring the association of different types of expenditure with the outcomes. Fourth, the Gelberg-Andersen Behavioral Model for Vulnerable Populations suggested a theory of change between determinants and health care access as well as utilization. Outcomes such as medication adherence would be worthy of investigation to reveal the extent to which financial burden affects utilization, which is further down the stream in the model. However, MEPS currently does not have such data available. Fifth, this study was also limited by the availability of locational data. Delays in medical care may not be entirely due to an ability to pay and delays may be related to the geographic location of the patients. Future studies should explore the effects of location. Finally, this study focused on the importance of incorporating consumer income into drug pricing. While this study has identified that patient income can play a critical role in patients’ process for seeking care, this study did not produce a specific pricing regime for incorporating patient income in pricing or propose an alternative threshold for cost-effectiveness. Future studies may be needed to devise such a pricing regime.

Despite the above limitations, the study contributed to a better understanding of the association between different measures of financial burden and health care access. It illustrated the importance of including income in policy considerations intended to balance value, access, and affordability. More specifically, income should be included in measures assessing the value of medications. When data become available, future research could further examine how a more accurate measure of financial burden such as the ratio of OOP expenditure to disposable income affects outcomes related to access and utilization.

## Author contributions

**Conceptualization:** Junling Wang.

**Data curation:** Xiaobei Dong, Chi Chun Steve Tsang.

**Formal analysis:** Xiaobei Dong, Chi Chun Steve Tsang.

**Funding acquisition:** Junling Wang.

**Investigation:** Xiaobei Dong, Chi Chun Steve Tsang.

**Methodology:** Xiaobei Dong, Chi Chun Steve Tsang, Junling Wang, Anoop Kotian, Jason Zeng, Michael Tran.

**Project administration:** Xiaobei Dong, Junling Wang.

**Software:** Chi Chun Steve Tsang, Xiaobei Dong.

**Supervision:** Junling Wang.

**Writing – original draft:** Xiaobei Dong, Chi Chun Steve Tsang.

**Writing – review & editing:** Xiaobei Dong, Anoop Kotian, Jason Zeng, Michael Tran, Junling Wang.
